# A Systematic Comparison of Data Selection Criteria for SMT Domain Adaptation

**DOI:** 10.1155/2014/745485

**Published:** 2014-02-11

**Authors:** Longyue Wang, Derek F. Wong, Lidia S. Chao, Yi Lu, Junwen Xing

**Affiliations:** Natural Language Processing & Portuguese-Chinese Machine Translation Laboratory, Department of Computer and Information Science, University of Macau, Macau, China

## Abstract

Data selection has shown significant improvements in effective use of training data by extracting sentences from large general-domain corpora to adapt statistical machine translation (SMT) systems to in-domain data. This paper performs an in-depth analysis of three different sentence selection techniques. The first one is cosine tf-idf, which comes from the realm of information retrieval (IR). The second is perplexity-based approach, which can be found in the field of language modeling. These two data selection techniques applied to SMT have been already presented in the literature. However, edit distance for this task is proposed in this paper for the first time. After investigating the individual model, a combination of all three techniques is proposed at both corpus level and model level. Comparative experiments are conducted on Hong Kong law Chinese-English corpus and the results indicate the following: (i) the constraint degree of similarity measuring is not monotonically related to domain-specific translation quality; (ii) the individual selection models fail to perform effectively and robustly; but (iii) bilingual resources and combination methods are helpful to balance out-of-vocabulary (OOV) and irrelevant data; (iv) finally, our method achieves the goal to consistently boost the overall translation performance that can ensure optimal quality of a real-life SMT system.

## 1. Introduction

The performance of SMT [1] system depends heavily upon the quantity of training data as well as the domain-specificity of the test data with respect to the training data. A well-known challenge is that the data-driven system is not guaranteed to perform optimally if the data for training and testing are not identically distributed. Thus, domain adaptation has been the promising study to boost the domain-specific translation from the model trained on mixture of in-domain and out-of-domain data.

One of the dominant approaches is to select suitable data for the target domain from a large general-domain corpus (general corpus), under the assumption that the general corpus is broad enough to cover some percentage of sentences that fall in the target domain. Then a domain-adapted machine translation system can be obtained by training on the selected subset (Axelrod et al. [2] defined it as *pseudo in-domain subcorpus*, which will also be adopted in this paper) instead of the entire data. Our work mainly focuses on these supplementary data selection approaches, which have shown significant improvements in the construction of domain-adapted SMT models. Models trained on such appreciated data will be benefited to improve the quality of word alignments. Secondly, extraction of a large amount of irrelevant phrase pairs can be prevented, and, thirdly, the estimation of reordering factors for target sentences can also be fairly optimized.

Data selection is often used for language model (LM) and translation model (TM) optimization. It generally comprises three processing stages in order to translate the domain-specific data *Q* using a general-domain parallel corpus *G* and a general-domain monolingual corpus *M* in target language.

*(1) Scoring.* The relevance of each sentence pair 〈*S*_*i*_, *T*_*i*_〉 in *G* to the target domain can be estimated by various similarity metrics, which can be uniformly stated as follows:

(1) $$\begin{array}{c} \text{Score}\left(S_{i},T_{i}\right)⟶\text{Sim}\left(V_{i},R\right), \end{array}$$

where the sentences in source *S*_*i*_, target *T*_*i*_, or both sides can be considered for similarity measuring; thus we uniformly define them as *V*_*i*_. And *R* is an abstract model to represent the target domain. These various applications of (1) will be detailed in Section 3.

*(2) Resampling.* Each scored sentence pair that is given a high weight value will be kept, otherwise will be removed from the pseudo in-domain subcorpus according to a binary filter function:

(2) $$\begin{array}{c} \text{Filter}\left(\text{Scor}{\text{e}}_{i}\right)=\{\begin{array}{cc} 1, & \text{Scor}{\text{e}}_{i}>\theta \\ 0, & \text{Others} \end{array} \end{array}$$

in which Score_*i*_ is the similarity value of the *i*th sentence pair 〈*S*_*i*_, *T*_*i*_〉 according to (1) and *θ* is a tunable threshold. It gives 1 for 〈*S*_*i*_, *T*_*i*_〉, with score higher than *θ*, and zero otherwise.

Note that the first two steps can also be applied to the selection of *M*, for the construction of the LM. The only difference is that (1) and (2) can only consider a sentence *L*_*i*_ of *M* in monolingual environment.

*(3) Translation.* After collecting a certain amount of 〈*S*_*i*_, *T*_*i*_〉 or 〈*L*_*i*_〉, TM and LM will be trained on these pseudo in-domain subsets.

A phrase-based SMT model, shown in (3), can be employed to obtain the best translation:

(3) Tbest=argmaxTPr⁡(T ∣ S)=argmaxT∑m=1Mλmhm(T,S),

where the *h*_*m*_(*t*, *s*) represents a feature function and *λ*_*m*_ is the weight assigned to the corresponding feature function. In general, the SMT system uses a total of eight features: an *n*-gram LM, two phrase translation probabilities, two lexical translation probabilities, a word penalty, a phrase penalty, and a linear reordering penalty [3–5].

As similarity measuring has shown a great impact on translation qualities, our goal is to find what kind of data selection model can better benefit the domain-specific translation effectively and robustly. Therefore, we systematically investigated and compared three state-of-the-art data selection criteria. The first two are the cosine tf-idf and perplexity-based criteria, which are techniques of information retrieval and language modeling for SMT. The third one is the edit-distance-based method, which is to be explored for the first time for this special task.

The results show that each individual criterion has the following natural pros and cons.

- Cosine tf-idf regards text as a bag of words and tries to retrieve similar sentences according to *word overlap*. Although it is helpful to reduce the out-of-vocabulary (OOV) words, this simple cooccurrence based matching will result in weakness of filtering irrelevant data (noise).
- Perplexity-based similarity metrics employ an *n*-gram LM, which considers not only the distribution of terms but also the collocation (*n*-gram *word order*). It works well in balancing the OOVs and noise by considering more factors. But its performance is sensitive to the quality of an in-domain LM as well as the quantity of pseudo in-domain subcorpus.
- Edit-distance-based method is much stricter than the former two criteria, because the factors of *words overlap*, *order*, and *position* are all comprehensively considered for similarity measuring. This seems to be able to find the most similar sentences, but it finally fails to outperform the general baseline in our experiments.

**Figure 1:**
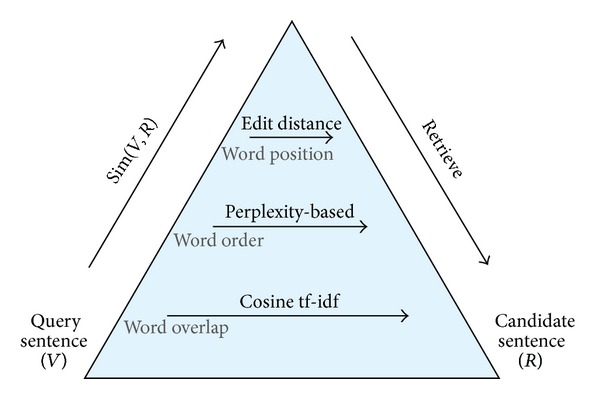
Data selection criteria pyramid.

The more factors are considered, the higher constraint degree of similarity criteria are. This can be depicted by a pyramid.  Figure 1 shows the comparative depths of different criteria: edit distance at the peak, followed by perplexity-based and the cosine tf-idf. The method would cover all the factors underneath. For instance, perplexity-based methods consider both word overlap and word order in similarity measuring. With considering more factors, the criterion at higher level is stricter than the ones below.

Although most SMT systems optimized by the presented methods can outperform the baseline system trained on general corpus (general baseline), their improvements are still either unclear or unstable (in Section 2). It is hard for these individual models to be applied in a real-life system; thus a combination approach is proposed as a trade-off. We combine all the presented (cosine tf-idf, perplexity-based, and edit-distance-based; criteria by performing linear interpolation at two levels: (i) *corpus level*, where we join the pseudo in-domain subcorpora selected by different similarity metrics at the resampling stage, and (ii) *model level*, where multiple models trained on pseudo in-domain subsets retrieved via different similarity metrics are combined together at translation stage. Since TM adaptation and LM adaptation may benefit each other [6], we combine these two adaptation approaches.

Comparative experiments were conducted using a large Chinese-English general corpus, where SMTs are trained and adapted to translate the in-domain sentences of the Hong Kong law statements. We measure the influence of different data selection methods on the final translation output. Using BLEU [7] as an evaluation metric, results indicate that our proposed approach successfully obtains a robust performance, which is still better than any single individual model as well as the baseline system. Although the edit-distance-based technique does not outperform the others in the individual setting, the selected data by this technique can complement the data retrieved by the other techniques, as demonstrated in the combined scenario. Although the edit-distance-based technique is the strictest one among the presented criteria, it fails to outperform others for the domain-specific translation task.

If a small in-domain corpus is available, it is good to use this to select pseudo in-domain data from general corpus as well as improve the current translation systems via combination methods. Thus we further combine the adapted system trained on pseudo in-domain data with the one trained on a small in-domain corpus using multiple paths decoding technique (as discussed in Section 2). Finally, we show that combining this small in-domain TM can further improve the best domain-adapted SMT system by up to 1.21 BLEU points and is also better than the general + in-domain system by at most 3.31 points.

The remainder of this paper is organized as follows. We firstly review the related work in Section 2. The proposed and other related similarity models are described in Section 3.  Section 4 details the configurations of experiments. Finally, we compare and discuss the results in Section 5 followed by the conclusions to end the paper.

## 2. Related Work

Researchers discussed the domain adaptation problems for SMT in various perspectives such as mining unknown words from comparable corpora [8], weighted phrase extraction [9], corpus weighting [10], and mixing multiple models [11–13]. Actually, data selection is one of the corpus weighting methods (there are data selection, data weighting and translation model adaptation) [14]. In this section, we will revisit the state-of-the-art selection criteria and our combination techniques.

### 2.1. Data Selection Criteria

The first selection criterion is the cosine tf-idf (term frequency-inverse document frequency) similarity, which comes from the realm of information retrieval (IR). Hildebrand et al. [15] applied this IR technique to select less but more similar sentences to the adaptation of TM and LM. They concluded that it is possible to adapt this method to improve the translation performance especially in the LM adaptation. Similar to the experiments described in this paper, Lü et al. [6] proposed resampling and reweighting methods for online and offline TM optimization, which are closer to a real-life SMT system. Furthermore, their results indicated that duplicated sentences can affect the translations. They obtained about 1 BLEU point improvement using 60% of total data. In this study, we still consider using duplicated sentences as a hidden factor for all the presented methods.

The second category is perplexity-based approach which can be found in the field of language modeling. This has been adapted by Lin et al. [16] and Gao et al. [17], in which perplexity is used to score text segments according to an in-domain LM. More recently, Moore and Lewis [18] derived the cross-entropy difference metric from a simple variant of Bayes rule. However, this is a preliminary study that did not yet show an improvement for MT task. The method was further developed by Axelrod et al. [2] for SMT adaptation. They also presented a novel bilingual method and compared it with other variants. The experimental results show that the fast and simple technique allows to discard over 99% of the general corpus resulted in an increase of 1.8 BLEU points. However, they tested various perplexity-based methods only using limited threshold in (2) settings, which may not reflect their overall performances.

In addition, the previous work often separately discussed related methods on TM [2] or LM [15]. But Lü et al. [6] pointed out that combining LM and TM adaptation approaches could further improve their performance. In this paper, we optimized both TM and LM via data selection methods.

The third retrieval model is not explicitly used for SMT but is still applicable to our scenario. Edit distance (ED) is a widely used similarity measure for example-based MT (EBMT), known as Levenshtein distance (LD) [19]. Koehn and Senellart [20] applied this method for convergence of translation memory (TM) and SMT. Leveling et al. [21] investigated different approximated sentence retrieval approaches (e.g., LD and standard IR) for EBMT. Both papers gave the exact formula of fuzzy matching. This inspires us to regard the metric as a new data selection criterion for SMT domain adaptation task. Good performance could be expected under the assumption that the general corpus is big enough to cover the very similar sentences with respect to the test data.

### 2.2. Combination Methods

The existing domain adaptation methods can be summarized into two broad categories: (i) *corpus level* by selecting, joining, or weighting the datasets upon which the models are trained and (ii) *model level* by combining multiple models together in a weighted manner [2]. In corpus level combination, the sentence frequency is often used as a weighting scheme. Hildebrand et al. [15] allowed duplicated sentences in selected dataset, which is a hidden bias to in-domain data. Furthermore, Lü et al. [6] gave each relevant sentence a higher integer weight in GIZA++ file. Mixture modeling approach is a standard technique in machine learning [22]. Foster and Kuhn [12] interpolated the multiple models together by performing linear and log-linear weights on the entries of phrase tables. Interpolation method is also often used in pivot-based SMT to combine standard and pivot models [23]. In order to investigate the best combination on these data selection criteria, we compare different combination methods at both corpus level and model level.

Furthermore, the additional in-domain corpora could also be used to enhance current TMs for domain adaptation. Linear interpolation (similar to combination at model level) could be applied to combine the in-domain TM with the domain-adapted TM. However, directly concatenating the phrase tables into one may lead to unpredictable behavior during decoding. In addition, Axelrod et al. [2] have concluded that it is more effective to use multidecoding method [24] than linear interpolation. Thus, we prefer to employ the confusion of multiple phrase tables at decoding model for this task.

## 3. Model Description

This section describes four data selection criteria, respectively, based on cosine tf-idf, perplexity, and edit distance as well as the proposed approach.

### 3.1. Cosine tf-idf

Each document *D*_*i*_ is represented as a vector (*w*_*i*1_, *w*_*i*2_,…, *w*_*in*_), and *n* is the size of the vocabulary. So *w*_*ij*_ is calculated as follows:

(4) $$\begin{array}{c} w_{ij}=\text{t}{\text{f}}_{ij}\times \log\left(\text{id}{\text{f}}_{j}\right) \end{array}$$

in which tf_*ij*_ is the term frequency (TF) of the *j*th word in the vocabulary in the document *D*_*i*_ and idf_*j*_ is the inverse document frequency (IDF) of the *j*th word calculated. The similarity between two texts is then defined as the cosine of the angle between two vectors. We implemented this with Apache Lucene (available at http://lucene.apache.org/) and it is very similar to the experiments described in [6, 15]. Suppose that *M* is the size of query set and *N* is the number of sentences retrieved from general corpus according to each query. Thus, the size of the cosine tf-idf based pseudo in-domain subcorpus is Size_Cos-IR_ = *M* × *N*.

### 3.2. Perplexity

Perplexity is based on the cross-entropy (5), which is the average of the negative logarithm of the word probabilities. Consider

(5) H(p,q)=−∑i=1np(wi)log⁡q(wi)=−1N∑i=1nlog⁡q(wi),

where *p* denotes the empirical distribution of the test sample. *p*(*w*_*i*_) = *n*/*N* if *w*_*i*_ appeared *n* times in the test sample of size *N*. *q*(*w*_*i*_) is the probability of event *w*_*i*_ estimated from the training set. Thus, the perplexity pp can be simply transformed as

(6) $$\begin{array}{c} \text{pp}=b^{H(p,q)}, \end{array}$$

where *b* is the base with respect to which the cross-entropy is measured (e.g., bits or nats). *H*(*p*, *q*) is the cross-entropy given in (5), which is often applied as a cosmetic substitute of perplexity for data selection [2, 18].

Let *H*_*I*_(*p*, *q*) and *H*_*O*_(*p*, *q*) be the cross-entropy of string *w*_*i*_ according to language model, LM_*I*_ and LM_*O*_ which are, respectively, trained by in-domain dataset *I* and general-domain dataset *G*. Considering the source (src) and target (tgt) sides of training data, there are three perplexity-based variants. The first is called basic cross-entropy given by

(7) $$\begin{array}{c} H_{I\text{-src}}\left(p,q\right). \end{array}$$

The second one is Moore-Lewis cross-entropy difference [18]:

(8) $$\begin{array}{c} H_{I\text{-src}}\left(p,q\right)-H_{G\text{-src}}\left(p,q\right) \end{array}$$

which tries to select the sentences that are more similar to *I* but different to others in *G*. All the above two criteria only consider the sentences in source language. Furthermore, Axelrod et al. [2] proposed a metric that sums cross-entropy difference over both sides:

(9) [HI-src(p,q)−HG-src(p,q)]  +[HI-tgt(p,q)−HG-tgt(p,q)].

The candidates with lower scores (obtained by (7), (8), and (9)) have higher relevance to target domain. The size of the perplexity-based pseudo in-domain subset Size_PP_ should be equal to Size_Cos-IR_. In practice, we perform SRILM toolkit (available at http://www.speech.sri.com/projects/srilm/) [25] to conduct 5-gram LMs with interpolated modified Kneser-Ney discounting [26].

### 3.3. Edit Distance

Given a sentence *s*_*G*_ from *G* and a sentence *s*_*R*_ from the test set or in-domain corpus, the edit distance for these two sequences is defined as the minimum number of edits, that is, symbol insertions, deletions, and substitutions, needed to transform *s*_*G*_ into *s*_*R*_. Based on Levenshtein distance or edit distance, there are several different implementations. We used the normalized Levenshtein similarity score (fuzzy matching score, FMS):

(10) $$\begin{array}{c} \text{FMS}=1-\frac{\text{LD}\left(s_{G},s_{R}\right)}{\mathrm{Max}\left(\left|s_{G}\right|,\left|s_{R}\right|\right)}, \end{array}$$

where |*s*_*G*_| and |*s*_*R*_| are the lengths *s*_*G*_ and *s*_*R*_ [20, 21]. In this study, we employed the word-based Levenshtein edit distance function. If there is a sentence of which its score exceeds a threshold, we would further penalize these sentences according to the whitespace and punctuations editing differences. The algorithm is implemented in map reduce to parallelize the process and shorten the processing time.

### 3.4. Proposed Model

For corpus level combination, we respectively perform GIZA++ toolkit (http://www.statmt.org/moses/giza/GIZA++.html) on pseudo in-domain subcorpora selected by different data selection methods. Then each sentence in different subcorpora can be weighted by modifying its corresponding occurrences in the GIZA++ file [6]. Finally, we combine these GIZA++ files together as a new one. Formally, this combination method can be defined as follows:

(11) $$\begin{array}{c} \alpha \;\mathrm{Cos}\;\text{IR}\left(S_{x},T_{x}\right),\\ \cup \beta \text{PP}\;\text{Based}\left(S_{y},T_{y}\right),\\ \cup \lambda \text{ED}\;\text{Based}\left(S_{z},T_{z}\right), \end{array}$$

where *α*, *β*, and *λ* are weights for sentences pairs (*S*_*x*_, *T*_*x*_), (*S*_*y*_, *T*_*y*_), and (*S*_*z*_, *T*_*z*_), which are selected by cosine tf-idf  (*Cos*⁡ IR), perplexity-based (PP Based), and edit-distance-based (ED Based) methods, respectively. Note that the number of sentence occurrence should be integer. In practice, we made *α*, *β*, and *λ* integer numbers and multiplied these weights with the occurrences of each corresponding sentence in GIZA++ file.

For model level combination, we perform linear interpolation on the models trained with the subcorpora retrieved by different data selection methods. The phrase translation probability $\phi (\bar{t}|\bar{s})$ and the lexical weight $p_{w}(\bar{t}|\bar{s},a)$ are estimated using (12) and (13), respectively, as follows:

(12) $$\begin{array}{c} \phi \left(\bar{t}\;|\;\bar{s}\right)=\sum_{i=0}^{n}\alpha_{i}\phi_{i}\left(\bar{t}\;|\;\bar{s}\right), \end{array}$$

(13) $$\begin{array}{c} p_{w}\left(\bar{t}\;|\;\bar{s},a\right)=\sum_{i=0}^{n}\beta_{i}p_{w,i}\left(\bar{t}\;|\;\bar{s},a\right), \end{array}$$

where *i* = 1,2, and 3 denoting the phrase translation probability and lexical weights trained on the subcorpora retrieved by *Cos*⁡ IR, PP Based, and ED Based model. $\bar{s}$ and $\bar{t}$ are the phrases in source and target language. *a* is the alignment information. *α*_*i*_ and *β*_*i*_ are the tunable interpolation parameters, subject to ∑*α*_*i*_ = ∑*β*_*i*_ = 1.

In this paper, we are more interested in the comparison of various data selection criteria, rather than the best combination method; thus we did not tune these weights in the above equations and gave them equal weights (At corpus level, *α* = *β* = *λ* = 1. At model level, *α*_*i*_ = *β*_*i*_ = 1/3) in experiments. Moreover, we will see that even these simple combinations can lead to an ideal improvement of translation quality.

## 4. Experimental Setup

### 4.1. Corpora

Two corpora are needed for the domain adaptation task. The general corpus includes more than 1 million parallel sentences comprising varieties of genres such as newswires (LDC2005T10), translation example from dictionaries, law statements, and sentences from online sources. The domain distribution of the general corpus is shown in Table 1. The miscellaneous part includes the sentences crawled from various materials and the law portion includes the articles collected from Chinese mainland, Hong Kong, and Macau, which have different laws. The in-domain corpus, development set, and test set are randomly selected (that are disjoined) from the Hong Kong law corpus (LDC2004T08). The size of the test set, in-domain corpus, and general corpus we used is summarized in Table 2.

In the preprocessing, the English texts are tokenized by Moses scripts (scripts are available at http://www.statmt.org/europarl/) [27] and the Chinese texts are segmented by NLPIR (available at http://ictclas.nlpir.org/) [28]. We removed the sentences of which length is more than 80. Furthermore, we only used the target side of data in general corpus as the general-domain LM training corpus. Thus, the target side of the pseudo in-domain corpus obtained by the previous methods (in Section 3) could be directly used for training adapted LM.

### 4.2. Systems Description

The experiments presented in this paper were carried out with the Moses toolkit [29], a state-of-the-art open-source phrase-based SMT system. The translation and the reordering model relied on “grow-diag-final” symmetrized word-to-word alignments built using GIZA++ [30] and the training script of Moses. The weights of the log-linear model were optimized by means of MERT [31].

A 5-gram language model was trained using the IRSTLM toolkit [32], exploiting improved modified Kneser-Ney smoothing and quantizing both probabilities and back-off weights.

### 4.3. Settings

For the comparison, totally, five existing representative data selection models, three baseline systems, and the proposed model were selected. The corresponding settings of the above models are as follows.

- Baseline: the baseline systems were trained with the toolkits and settings as described in Section 4.2. The in-domain baseline (IC-baseline) and general-domain baseline (GC-baseline) were, respectively, trained on in-domain corpus and general corpus. Then a combined baseline system (GI-baseline) was created by passing the above two phrase tables to the decoder and using them in parallel.
- Individual model: as described in Sections 3.1, 3.2, and 3.3, the individual models are cosine tf-idf  (Cos-IR) and fuzzy matching scorer (FMS) which is an edit-distance-based (ED-Based) instance as well as three perplexity-based (PP-Based) variants: cross-entropy (CE), cross-entropy difference (CED), and bilingual cross-entropy difference (B-CED).
- Proposed model: as described in Section 3.4, we combined Cos-IR, PP-Based, and ED-Based methods at corpus level (named *i*TPB-C) and model level (named *i*TPB-M).

The test set, development set [6, 15], and in-domain corpus [2, 18] can be used to select data from general-domain corpus. The first strategy is under the assumption that the input data is known before building the models. But in a real case, we do not exactly know the test data in advance. Therefore, we use the development set (dev. strategy) and additional in-domain corpus (in-domain strategy) which are identical to the target domain to select data for TM and LM adaptation.

## 5. Results and Discussions

For each method, we used the *N*-bests selection results, where *N* = {20 K, 40 K, 80 K, 160 K, 320 K, 640 K} sentence pairs out of the 1.1 M from the general corpus (roughly 1.75%, 3.5%, 7.0%, 14.0%, 28.0%, and 56% of general-domain corpus where *k* is short for thousand and *m* is short for million). In this section, we firstly discuss three PP Based variants and then compare the best one with other two models: Cos-IR and FMS. After that, combined model and the best individual are compared. Finally, the performance is further improved by a simple but effective system combination method.

### 5.1. Baselines

The baseline results (in Table 3) show that a translation system trained on the general corpus outperforms the one trained on the in-domain corpus over 2.85 BLEU points. The main reason is that general corpus is so broad that the OOVs are much lesser. Combining these two systems could further increase the GC-baseline by 1.91 points.

### 5.2. Individual Model

**Figure 2:**
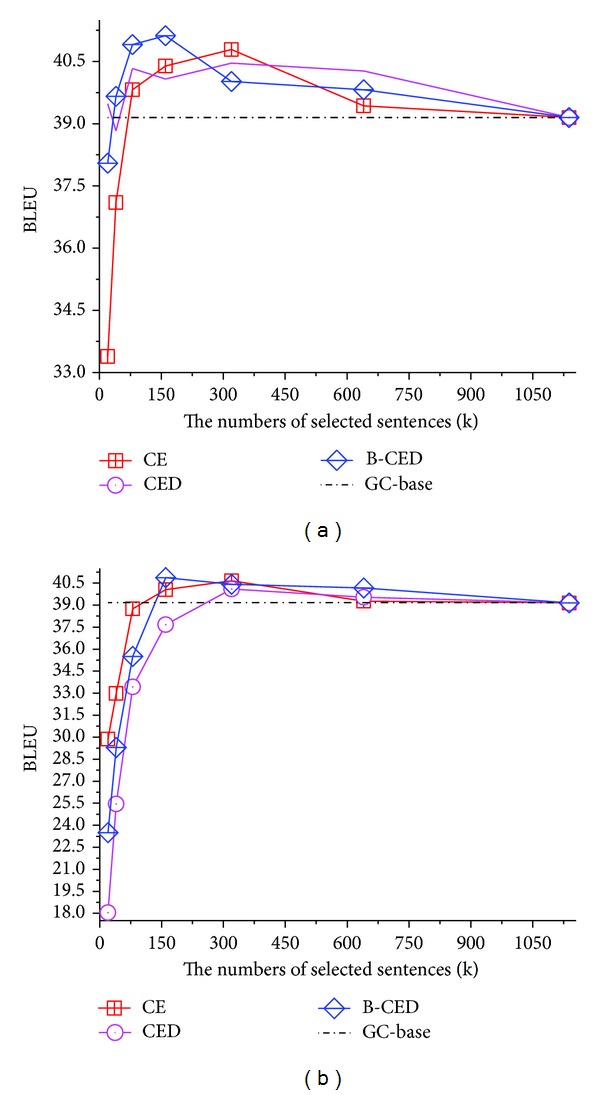
BLEU scores via perplexity-based data selection methods with dev. (a) and in-domain (b) strategies.

The curves in Figure 2 show that all the three PP Based variants, that is, CE, CED, and B-CED, could be used to train domain-adapted SMT systems. Using less training data, they still obtain better performance than GC-baseline. CE is the simplest PP Based one and improves the GC-baseline by at most 1.64 points when selecting 320 K out of 1.1 M sentences. Although CED gives bias to the sentences that are more similar to the target domain, its performance seems unstable with dev. strategy and the worst with in-domain strategy. This indicates that it is not suitable to randomly select data from general corpus for training LM_*O*_ (in Section 3.2). Similar to the conclusion in [2], B-CED achieves the highest improvement with least selected data among the three variants. It proves that bilingual resources are helpful to balance OOVs and noise. However, when using in-domain corpus to select pseudo in-domain subcorpora, their trends are similar but worse. They have to enlarge the size of selected data to perform nearly as well as the results under dev. strategy. Therefore, PP Based approaches may not work well for a real-life SMT application.

**Figure 3:**
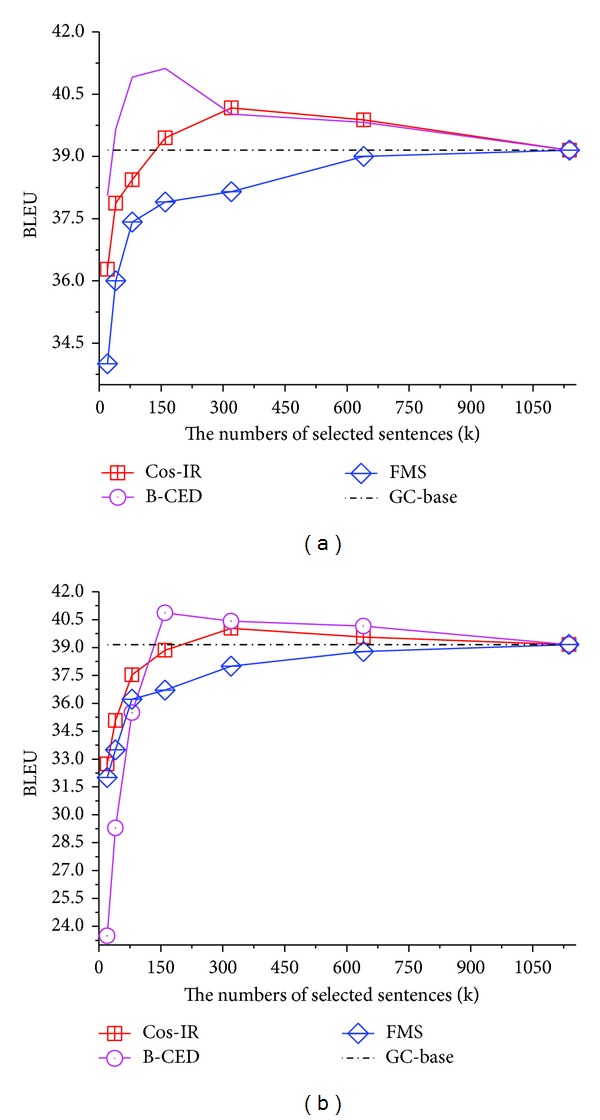
BLEU scores via different data selection methods with dev. (a) and in-domain (b) strategies.

Next we use B-CED (the best one among PP Based variants) to compare with other selection criteria. As shown in Figure 3, Cos-IR improves by at most 1.02 (dev) and 0.88 (in-domain) BLEU points using 28% data of the general corpus. The results approximately match with the conclusions given by [6, 15], showing that keywords overlap (in Figure 1) plays a significant role in retrieving sentences in similar domains. However, it still needs a large amount of selected data (more than 28.0%) to obtain an ideal performance. The main reason may be that the sentences including same keywords still may be irrelevant. For example, two sentences share the same phrase “*according to the article*,” but one sentence is in the legal domain and the other is from news. Figure 3 also shows that FMS fails to outperform the GC-baseline system even it is much stricter than other criteria. When adding word position factor into similarity measuring, FMS tries to find highly similar sentences on length, collocation, and even semantics. But it seems that our general corpus is not large enough to cover a certain amount of FMS-similar sentences. With the size of general or in-domain corpus increases, it may benefit the translation quality, because FMS still works better than IC-baseline, which proves its positive impact on filtering noise.

Among the three presented criteria, PP Based can achieve the highest BLEU with considering an appropriate amount of factors for similarity measuring. However, the curves show that it depends heavily upon the threshold *θ* in (2). Selecting more or less pseudo in-domain data will lead to the performance dropping sharply. Instead, Cos-IR works steadily and robustly with either and both strategies, but its improvements are not clear. Thus any single individual model cannot perform well on both effectiveness and robustness.

### 5.3. Combined Model

From Figure 3, we found that each individual model peaks between 80 K and 320 K. Thus, we only selected the top *N* = {80 K, 160 K, 320 K} for further comparison. We combined Cos-IR and FMS as well as B-CED and assigned equal weights to each individual model at both corpus and model levels (as described in Section 3.4). The translation qualities via *i*TPB are shown in Table 4.

At both levels, *i*TPB performs much better than any single individual model as well as GC-baseline system. For instance, *i*TPB-C has achieved at most 3.89 (dev) and 2.72 (in-domain) improvements than the baseline system. Also the result is still higher than the best individual model (B-CED) by 1.92 (dev) and 0.91 (in-domain). This shows a strong ability to balance OOV and noise. On the one hand, filtering too much unmatched words may not sufficiently address the data sparsity issue of the SMT model; on the other hand, adding too much of the selected data may lead to the dilution of the in-domain characteristics of the SMT model. However, combinations seem to succeed the pros and reduce the cons of the individual model. In addition, the performance of *i*TPB does not drop sharply when changing the threshold in (2) or the strategies. This shows that combined models are both stable and robust to train a state-of-the-art SMT system. We also prove that model level combination works better (up to +1 BLEU) than corpus level combination with the same settings.

### 5.4. System Combination

In order to advantageously use all available data, we also combine the in-domain TM with pseudo in-domain TM to further improve the translation quality. We used multiple paths decoding technique as described in Section 2.2.

Table 5 shows that the translation system trained on a pseudo in-domain subset selected with individual or combined models can be further improved by combining with an in-domain TM. The *i*TPB-M+I system comprising two phrase tables trained on *i*TPB-M based pseudo in-domain subset and in-domain corpus still works best. This tiny combined system is at most 3.31+ (dev) and 1.47+ (in-domain) points better than the GI-baseline system, 8.07+ (dev) and 6.23+ (in-domain) points better than the in-domain system alone.

## 6. Conclusions

In this paper, we analyze the impacts of different data selection criteria on SMT domain adaptation. This is the first time to systematically compare the state-of-the-art data selection methods such as cosine tf-idf and perplexity. Among the revisited methods, we consider edit distance as a new similarity metric for this task. The in-depth analysis can be very valuable to other researchers working on data selection.

Based on the investigation, a combination approach to make use of those individual models is extensively proposed and evaluated. The combination is conducted at not only the corpora level but also the models level where domain-adapted systems trained via different methods are interpolated to facilitate the translation. Five individual methods, Cos-IR, CE, CED, B-CED, and FMS; three baseline systems and GC-baseline, IC-baseline, and GI-baseline, as well as the proposed method are evaluated on a large general corpus. Finally, we further combined the best domain-adapted system with the TM trained on in-domain corpus to maximize the translation quality. Empirical results reveal that the proposed model achieves a good performance in terms of robustness and effectiveness.

We analyze the results from three different aspects.

- Translation quality: the results show a significant performance of the most methods especially for *i*TPB. Under the current size of datasets, considering more factors in similarity measuring may not benefit the translation quality.
- Noise and OOVs: it is a big challenge to balance them for single individual data selection model. However bilingual resources and combination methods are helpful to deal with this problem.
- Robustness and effectiveness: a real-life system should achieve a robust and effective performance with in-domain strategy. Only *i*TPB obtained a consistently boosting performance.

Finally, we can draw a composite conclusion that (*a* > *b* means that *a* is better than *b*)

(14) $$\begin{array}{c} i\text{TPB}>\text{PP}\;\text{Based}>\text{Cos-IR}>\text{Baseline}>\text{FMS}. \end{array}$$

## Figures and Tables

**Table 1:** Proportions of domains of general corpus.

Domain	Sent. number	%
News	279,962	24.60
Novel	304,932	26.79
Law	48,754	4.28
Miscellaneous	504,396	44.33

Total	1,138,044	100.00

**Table 2:** Corpora statistics.

Data Set	Lang.	Sentences	Tokens	Av. len.
Test set	EN	2,050	60,399	29.46
	ZH		59,628	29.09

Dev. set	EN	2,000	59,732	29.26
	ZH	2,000	59,064	29.07

In-domain	EN	43,621	1,330,464	29.16
	ZH		1,321,655	28.97

Training set	EN	1,138,044	28,626,367	25.15
	ZH		28,239,747	24.81

**Table 3:** BLEU via general-domain and in-domain corpus.

Baseline	Sentences	BLEU
GC-baseline	1.1 M	39.15
IC-baseline	45 K	36.30
GI-baseline		41.06

**Table 4:** Translation results of *i*TPB at different combination levels.

Methods	Sent.	BLEU (dev. set)	BLEU (in-domain set)
B-CED	80 K	40.91	35.50
	160#x2009;K	**41.12**	39.47
	320#x2009;K	40.02	**40.98**

*i*TPB-C	80#x2009;K	42.25	39.39
	160#x2009;K	**43.04**	**41.87**
	320#x2009;K	42.42	40.44

*i*TPB-M	80#x2009;K	42.93	40.57
	160#x2009;K	43.65	41.95
	320#x2009;K	**43.97**	**42.21**

**Table 5:** Results of combination models.

Methods	Sent.	BLEU (dev. set)	BLEU (in-domain set)
GI-baseline		**41.06**	
IC-baseline	45 K	36.30	
B-CED+I	80 K	41.05	35.80
	160 K	41.14	39.54
	320 K	**41.67**	**40.67**
*i*TPB-C+I	80 K	42.85	41.45
	160 K	43.48	41.88
	320 K	**43.63**	**41.98**
*i*TPB-M+I	80 K	43.33	41.83
	160 K	44.13	42.43
	320 K	**44.37**	**42.53**
